# Heart Rate Variability and Cognitive Function Following a Multi-Vitamin and Mineral Supplementation with Added Guarana (*Paullinia cupana*)

**DOI:** 10.3390/nu7010196

**Published:** 2014-12-31

**Authors:** Laura Pomportes, Karen Davranche, Ioanna Brisswalter, Arnaud Hays, Jeanick Brisswalter

**Affiliations:** 1 Laboratory of Human Motricity, Education, Sport and Health (EA 6309), University of Nice Sophia Antipolis, Nice 06205, France; E-Mails: laurapomportes@hotmail.fr (L.P.); withoutconcept@gmail.com (I.B.); 2 Aix Marseille University, CNRS, LPC UMR 7290, FR 3C FR 3512, Marseille 13000, France; E-Mail: karen.davranche@univ-amu.fr; 3 National Institute for Sport Expertise and Performance, Paris 75012, France; E-Mail: arnaud.hays@gmail.com; 4 Creps Sud-Est, Aix en Provence 13080, France

**Keywords:** guarana, reaction time, decision-making, caffeine, heart rate variability

## Abstract

The aim of this study was to assess cognitive performance and heart rate variability (HRV) following the ingestion of either a multi-vitamin-mineral preparation supplemented with 300 mg guarana (Ac); a caffeine supplement (C) or a placebo supplement (Pl). Fifty-six subjects took part in a randomized, double-blind crossover design, consisting of three experimental sessions ran on a different day. Cognitive performance was assessed using a go/no-go task and a simple reaction time (SRT) task. HRV was assessed in the time domain (RMSSD) and in the frequency domain (HF) and cognitive tasks were performed before ingestion, 15 min after ingestion and then every 15 min over the course of 3 h. Responses were faster (without change in accuracy) when the go/no-go task was performed between 30 and 90 min after ingestion of Ac (4.6% ± 0.8%, *p* < 0.05). No effect was observed on SRT task. A significant decrease in HRV was observed during the first hour under C and Pl, whereas HRV remained stable under Ac. The results suggest that the ingestion of a multi-vitamin-mineral with added guarana improves decision-making performance and is accompanied by a stable autonomic nervous system regulation during the first hour.

## 1. Introduction

Guarana (*Paullinia Cupana)* is a climbing plant of the maple family. It is a rain forest vine that was domesticated in the Amazon for its fruits, which are rich in caffeine. Despite its increasing usage as a dietary constituents by athletes to support training or improve performance, guarana’s specific behavioral effects have received little attention until recently. Currently there has been a growing interest in the beneficial effects of guarana, which is supported by a number of experimental studies in humans and rodents. Guarana has been reported to delay physical and mental cancer-related fatigue [1,2], improve cognitive performance [3,4,5,6,7], have positive effects on mood [6,7] and facilitate weight loss [8]. Guarana administration in rats reduced total food intake, carcass fat content and decreased plasma lactate concentrations in trained rats [9]. Guarana’s may also be used as an antibacterial agent and has antioxidant properties [10].

The psychoactive properties of a single dose of guarana extract (75 mg) have been initially observed by Kennedy and colleagues in healthy adults [5]. Authors reported improvements in memory performance (*i.e.*, long-term storage system or secondary memory) and in response speed (albeit accompanied with a decrease in accuracy, suggesting a speed-accuracy trade-off). Using different doses of guarana extract (*i.e.*, 37.5 mg, 75 mg, 150 mg and 300 mg), Haskell and colleagues [6] confirmed an improvement of cognitive performance and highlighted changes in mood. Interestingly, there is an increase in self-rated “alertness” following the highest dose of guarana and an increase in self-rated “contentment” following all doses.

The psychoactive properties of guarana have often been attributed to the high concentration of caffeine contained in its seeds. However, the results of these latest studies [5,6] show that psychoactive effects can be observed using a 75 mg dose of guarana, which contains a level of caffeine considered too low (9 mg) to produce positive effects. These findings suggest that cognitive performance changes induced by guarana cannot be solely attributable to guarana’s caffeine content. Evidence suggests that other components, such as flavonoids [11] or other potentially psychoactive components, including saponins and tannins [12,13], may contribute to cognitive function changes under guarana. Scholey and Haskell [11] suggested that these psychoactive effects could be attributable to modulation of guarana’s caffeine content by other guarana components or by direct effects of noncaffeine constituents contained in guarana. To date, the relative contribution of caffeine and guarana is unclear and deserves further investigations to disentangle their psychoactive effects. The first aim of this study was to compare the effects of a multi-vitamin-mineral preparation supplemented with 300 mg guarana on cognitive function with an equivalent pure caffeine dose over the course of 3 h.

The efficacy of guarana supplementation associated with vitamins/minerals in terms of cognitive performance has been investigated more recently. Scholey and colleagues [14] were the first to bring to evidence that a multivitamin-mineral preparation containing guarana improved cognitive performance in humans and reduced mental fatigue associated with sustained mental effort. Following the administration of a single dose of a vitamin/mineral/guarana food supplement, an improvement in performance, both in terms of speed and accuracy, as well as an attenuation of subjective mental fatigue have been observed during a sustained cognitive task (*i.e.*, Rapid Visual Information Processing Task, (RVIP) (During the RVIP task, subjects are requested to detect target sequences of digits and respond as quickly as possible by pressing a key response.)) [3]. More recently, Scholey and colleagues [7] evaluated the neurocognitive effects of multivitamin supplementations with and without guarana. The authors examined the neural substrates of guarana-induced effects using an fMRI protocol during a RVIP task and an Inspection Time (IT) activation task (During the IT task, subjects are requested to discriminate the longest of two lines displayed for a variable duration.). Results showed that both multivitamin treatments increased activation in areas involved in cognitive tasks, but intriguingly the activation was greater in the multivitamin with guarana condition.

Despite similar behavioral effects, it is not clear if the mechanisms of action responsible for the psychoactive effect of guarana on cognitive performance are similar with those described for caffeine [3]. The effect of caffeine on cognition is well documented; it has been shown that, caffeine alters attention and modifies the neural activity in cortical areas involved in the perceptual analysis of relevant stimulus information, e.g., [15]. However, the central nervous system does not seem to develop a great tolerance to caffeine and psychoactive effects can be accompanied by potential side effects, such as anxiety or nervousness [16], generated palpitations and arrhythmia. Because guarana contains caffeine, some similar potential side effects including psycho-physiological disturbances should be considered. Interactions between neural states and cognitive performance are a traditional topic of psychological research. Classical concepts from motivational psychology relate cognitive improvement with neural activation changes [17]. Within this framework, recent researchers have studied this relationship through the analysis of the autonomic nervous systems’ regulation assessed by heart rate variability (HRV) [18,19,20]. According to Porges’ [21] model, it could be predicted an association between higher resting levels of cardiac vagal tone and improved cognitive capacity [20]. Since HRV changes could represent activation [18] as well as anxiety [22], it could reflect, following ingestion, either positive or negative effects of guarana on cognition. To date, the effects of guarana on autonomic function remains unknown; the second aim of this study was to investigate potential side effects of guarana on the autonomic nervous systems’ regulation through the analysis of HRV over the course of 3 h.

## 2. Experimental Section

### 2.1. Participants

Fifty-six subjects (32 males and 24 females) volunteered to participate in the study after an Internet announcement of the research project on the university website. The mean age of the males was 27.7 years (range: 19–45 years, body mass (74.1 ± 5.7) kg, height (179 ± 3) cm) and of the females was 29.5 years (range: 18–42 years, body mass (65.6 ± 3.6) kg, height (166 ± 2) cm). All of the participants were caffeine consumers (range: 200–500 mg per day). Exclusion criteria comprised severe physical diseases, psychiatric disorders, chronic alcohol consumption, as well as the use of psychoactive drugs or medication affecting the cardiovascular system. The procedures followed were in accordance with the Declaration of Helsinki 1975, revised Hong Kong 1989. The local ethics committee (Ile-de-France VII, Saint Germain en Laye, France) reviewed and approved the study before its initiation and all subjects gave their informed written consent before participation.

### 2.2. Procedure

This study used a randomized, double blind, experimental design. Subjects were required to report to the laboratory for testing on three separate sessions with at least 48 h between each. Each subject was his own control, thus this study included three paired treatments: a vitamin/mineral/guarana supplement (Ac, *n* = 56); a caffeine supplement (C, *n* = 56); and a placebo supplement (P, *n* = 56). The three conditions were prepared by an independent laboratory and presented to the subjects mixed with water in a standardized blind conditioning. The vitamin/mineral/guarana complex was a commercially available effervescent tablet, Isoxan Actiflash^®^ (Menarini, NHS, Rungis, France). The constituents are shown in Table 1. Caffeine contains 100 mg of caffeine (Natrol^®^, Biovéa, Le Mans France), which corresponds to an equivalent dose to that contained in the vitamin/mineral/guarana tablet. All subjects were tested individually in a quiet room and the experimental session began after 15 min of rest. They were required to avoid any kind of exercise or consume any alcohol for 24 h prior to the experimental session. At the beginning of the session, subjects had to complete a self-administered questionnaire to assess overnight caffeine abstinence according to Hughes *et al.* [23] study. All assessments were done in the afternoon approximately at the same time (2–6 pm).

Cognitive tasks were performed before ingestion, 15 min after ingestion, and then every 15 min over the course of 3 h. Cognitive task duration was about 8 min. Before each cognitive task, an electrocardiogram (ECG) was recorded during 5 min using a Biopac 150 system (Biopac Systems Inc., Goleta, CA, USA). All measures before ingestion were used as a baseline value for each day.

**Table 1:** Contents of the vitamin/mineral tablets/guarana, Isoxan Actiflash^®^ effervescent tablets.

Active	Units	Per Day	European RDA	
			Males	Females
Vitamin C	180 mg	180 mg	90 mg	75 mg
Vitamin B1	0.705 mg	0.705 mg	1.2 mg	1.1 mg
Vitamin B2	0.798 mg	0.798 mg	1.3 mg	1.1 mg
Vitamin B3	9005 mg	9005 mg	16 mg	14 mg
Vitamin B6	1003 mg	1003 mg	1.7 mg	1.5 mg
Zinc	7490 mg	7490 mg	11 mg	8 mg
Guaraná ES	300 mg	300 mg	NA	NA
Ginseng ES	100 mg	100 mg	NA	NA

RDA (Recommended Dietary Allowances) are provided for males and females age ranging from 19 years to 70 years (European Food Safety Authority, 2010).

### 2.3. Cognitive Performance

Cognitive performances were assessed using two different tasks: a go/no-go task and a simple reaction time (SRT) task.

#### 2.3.1. Go/No-Go Task

Stimuli were presented in white on a black background. Stimuli consisted of an H or an S. Participants were instructed to press a button if the stimulus was an H, and not to respond if the stimulus was an S. Both stimuli were equiprobable. Stimuli remained on the screen until a response was made or until 1200 ms had elapsed. There was an interval of 400–600 ms before the start of the next trial.

#### 2.3.2. Simple Reaction Time (SRT) Task

The SRT task was measured from impulses recorded on a handgrip while the subject was seated. The subject was instructed to hold the handgrip in his/her preferred hand and to place his/her thumb on a button. SRT was calculated as the time required by the subject to remove his/her thumb from the button. This device was connected to a microcomputer with a sampling frequency of 120 Hz. The luminous stimuli appeared in the center of a screen and were separated by a variable foreperiod varying from 3–5 s Mean SRT and standard deviation were calculated for 40 trials and SRT under 160 ms was considered as an error (anticipated responses).

### 2.4. Heart Rate Measure

During the ECG assessment periods, participants were instructed to sit relaxed with hands on thighs without speaking or moving, and to breathe regularly. Two active electrodes (Ag/AgCl) were placed at the right mid-clavicle and the lowest left rib. Respiration was monitored with the same Biopac system using a piezoelectric thoracic belt. The QRS-signal waveform (R-R signal) was sampled at the resolution of 1 ms. The R-R intervals (*i.e.*, the length of time between the R peaks of consecutive QRS complexes) were calculated and checked for artifacts. The root mean square difference of successive normal R-R intervals (RMSSD) was calculated for the 5 min of recorded data. A fixed linear resampling frequency of 1024 equally spaced points per 3 min period was used. Power density in the HF (0.15–0.50 Hz) band was calculated for every three-minute spectrum by integrating the spectral power density within the frequency band [18,19,20]. The Respiratory Rate (RR) measure enabled us to conduct analyses with RR controlled since the central frequency of the HF component has been shown to be highly correlated with strain gauge measures of respiration [24].

### 2.5. Statistics

Changes from baseline scores for all dependent variables were calculated in contrast with pre-dose performance. Each dependent variable was analyzed using a repeated measures analysis of variance (ANOVA), including conditions (vitamin/mineral/guarana, Ac; caffeine, C; and placebo, Pl) × time (8 periods over the course of 3 h). The effect size was calculated using eta squared(eta2) values. Follow-up analyses were conducted using simple effect tests using Tukey’s procedure. For analyses involving time, if the sphericity assumption was violated, then Greenhouse-Geisser [25] conservative degrees-of-freedom adjustments were applied and critical p values were corrected [26]. The data was analyzed using the Statistica 7.1 program (StatSoft, Maisons-Alfort, France)

## 3. Results

### 3.1. Cognitive Performance

Cognitive performance values at baseline and among times are represented in Table 2. No significant difference was found between male and female responses in this study therefore data is presented for the whole population.

**Table 2:** Descriptive statistics for cognitive performance and Heart Rate variability as a function of experimental condition (vitamin/mineral/guarana, Ac; caffeine, C; and placebo, Pl) at baseline and every 15 min over the course of 3 h.

Go/No-Go	Condition		Time (min)								
			0	15	30	45	60	90	120	150	180
reaction time (ms)	Ac	mean	329	329	317 *	314 *^,§^	315 *^,§^	325 *^,§^	345	347 *	349 *
		SD	16	13	24	18	25	19	14	17	20
	Pl	mean	330	331	334	339	345	352 ^‡^	359 ^‡^	358 ^‡^	358 ^‡^
		SD	18	25	32	46	33	25	26	21	13
	C	mean	327	329	329	333	335	340	350 ^†^	355 ^†^	356 ^†^
		SD	17	19	28	32	29	22	20	19	16
Go/no-go errors (%)	Ac	mean	1	0.6	0.8	0.7	1.3	0.5	0.7	1.2	0.5
		SD	0.7	0.7	0.4	1.5	0.3	0.6	0.4	0.8	0.8
	Pl	mean	1	0.6	0.0	0.9	0.6	0.5	0.4	0.8	0.6
		SD	0.5	0.0	1.0	0.3	0.3	0.4	0.4	0.3	0.5
	C	mean	0	0.6	0.4	0.8	0.9	0.5	1.3	1.0	0.6
		SD	0.6	0.3	0.7	0.9	0.3	0.5	0.4	0.5	0.7
Simple reaction time (ms)	Ac	mean	230	224	227	256 *	247 *	251 *	250 *	242 *	246 *
		SD	11	9	17	13	18	13	10	12	14
	Pl	mean	228	233	242	264 ^‡^	253 ^‡^	259 ^‡^	249 ^‡^	245 ^‡^	253 ^‡^
		SD	12	18	22	32	23	17	18	15	9
	C	mean	227	227	233	258 ^†^	248 ^†^	253 ^†^	248 ^†^	242 ^†^	248 ^†^
		SD	12	13	19	22	20	15	14	13	12
RMSSD (ms)	Ac	mean	64.1	66.6	63.3	63.6 ^§^	60.6 *^,^^§^	60.7 *^,^^§^	57.3 *	56.2 *	54.5 *
		SD	8.9	10.5	12.7	4.3	4.1	5.8	5.7	7.4	7.8
	Pl	mean	67.0	66.2	61.4 ^‡^	59.8 ^‡^	56.5 ^‡^	54.8 ^‡^	54.1 ^‡^	53.3 ^‡^	54.6 ^‡^
		SD	10.2	11.6	15.5	8.3	2.4	4.5	2.2	3.8	11.2
	C	mean	65.2	66.0	64.3	61.4 ^†^	58.2 ^†^	57.4 ^†^	55.4 ^†^	54.5 ^†^	54.3 ^†^
		SD	9.5	11.0	14.0	6.3	3.3	5.1	8.9	5.6	9.4
HF (n.u.)	Ac	mean	41.1	40.7	40.6	39.7 ^§^	38.7 *^,^^§^	36.9 *	35.5 *	36.3 *	35.6 *
		SD	2.8	4.5	5.8	8.1	5.9	8.4	2.1	5.6	3.7
	Pl	mean	41.6	40.5	36.6 ^‡^	35.6 ^‡^	35.5 ^‡^	35.4 ^‡^	35.8 ^‡^	36.1 ^‡^	35.8 ^‡^
		SD	9.8	3.2	3.7	6.3	3.2	3.6	5.1	4.1	4.8
	C	mean	41.4	40.5	39.9	37.5 ^†^	36.6 ^†^	36.0 ^†^	35.9 ^†^	36.5 ^†^	36.0 ^†^
		SD	6.2	3.8	4.7	7.1	4.5	6.0	3.6	3.9	5.1

* When a difference was significant with baseline for Ac condition; ^†^ when a difference was significant with baseline for C condition; ^‡^ when a difference was significant with baseline for Pl condition; ^§^ when a difference was significant between Ac and C condition.

#### 3.1.1. Go/No-Go Task

**Figure 1:**
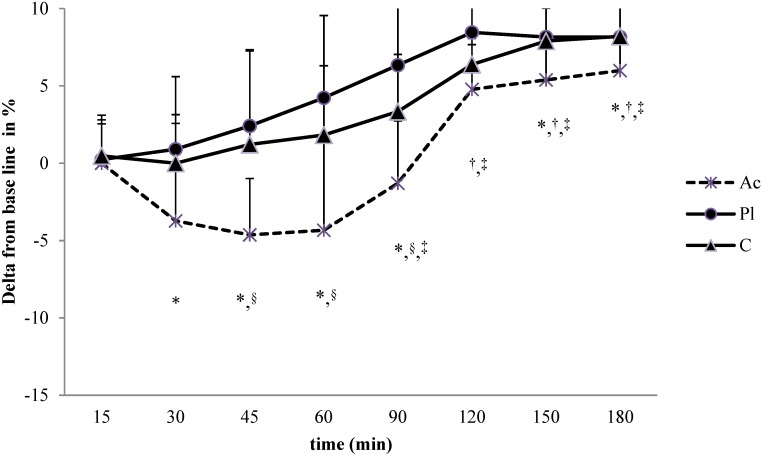
Change in reaction time performance (in percentage from baseline values) during the go/no-go task performed under vitamin/mineral/guarana (Ac), caffeine (C) and placebo (Pl) supplementation over the course of 3 h. * When a difference was significant with baseline for Ac condition; ^†^ when a difference was significant with baseline for C condition; ^‡^ when a difference was significant with baseline for Pl condition; ^§^ when a difference was significant between Ac and C condition.

There was a significant main effect of time (*p* < 0.05; eta2 = 0.42) and a significant interaction between time and condition (*p* < 0.01; eta2 = 0.63) on mean reaction time (RT) during the go/no-go task (Figure 1). For Ac, faster RT was observed from the 30th minute to the 90th minute with a significant difference between Ac and C conditions (maximal difference between C and Ac at the 60th minute: 8.5% ± 0.7%, *p* < 0.05) (Figure 1). In all conditions a significant decrease in performance was observed at the end of the experiment (respectively, for Pl, C and Ac conditions: −8.1% ± 2.6% *vs.* −8.2% ± 1.9% *vs.* −5.9% ± 1.7%). This impairment became significant at the 90 min for Pl condition (−6.3% ± 1.8%, *p* < 0.05) ) and at the 120th minute for C condition (−6.5% ± 2.2%, *p* < 0.05), whereas it occurred only at the 150th minute for Ac condition (−5.4% ± 1.1%, *p* < 0.05). No significant effect was found on errors.

#### 3.1.2. Simple Reaction Time (SRT) Task

A significant effect of the time spent on the task was observed on mean RT (*p* < 0.001 eta2 = 0.58) and no interaction was observed between time and condition. Reaction time lengthens with time on task in all conditions after 45th minute (respectively, for Ac, C and Pl at 45th minute: 11.45% ± 1.8% *vs.* 13.6% ± 2.5% *vs.* 15.8% ± 2.1%, *p* < 0.05). No further significant changes were observed. No significant effect of time or condition was observed on anticipated responses.

### 3.2. Heart Rate Variability (HRV)

**Figure 2:**
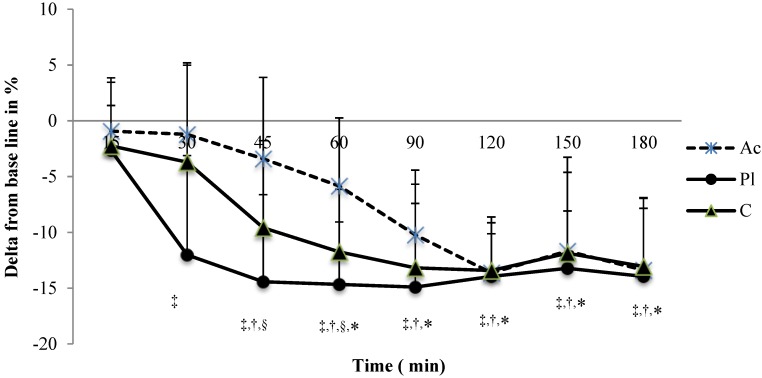
Graphic representation of change from baseline scores (in %) in the High Frequency (HF) band over the course of 3 h. * When a difference was significant with baseline for Ac condition; ^†^ when a difference was significant with baseline for C condition; ^‡^ when a difference was significant with baseline for Pl condition; ^§^ when a difference was significant between Ac and C condition.

Heart Rate variability values at baseline and among times are represented in Table 2. A significant interaction was observed between time and condition on both RMSSD and HF values (respectively, *p* = 0.01, eta2 = 0.68 and *p* = 0.01, eta2 = 0.63). In all conditions, a decrease in RMSSD was observed at the end of the experiment (respectively, for Pl, C and Ac conditions: −18.5% ± 3.8%; −16.8% ± 2.9% and −15.9% ± 2.9%). When compared with baseline, RMSSD values decreased at the 30th minute for Pl (−8.3% ± 2.4%, *p* < 0.05) and at the 45th minute for C (−5.9% ± 1.1%, *p* < 0.05), whereas it remained stable for Ac until the 60th minute (−5.1% ± 2.8%, ns). Furthermore, RMSSD values were significantly lower for C than for Ac between the 45th and the 90th minute (the maximal difference was observed at the 45th minute: 5.3% ± 0.5%, *p* < 0.05). A similar decrease was observed for HF values. After the 30th minute, the decline was maximal for both Pl (−12.2% ± 2.1%, *p* < 0.05) and C (−9.6% ± 1.8%, *p* < 0.05) and occurred later, at the 60th minute, for Ac condition (−5.8% ± 1.4%, *p* < 0.05). A significant difference was observed between C and Ac conditions between the 45th and the 60th minute (mean difference between C and Ac, respectively, at the 45th and the 60th minute: 6.1% ± 1.1% and 6.3% ± 0.9%, *p* < 0.05) (Figure 2).

## 4. Discussion

This study examined cognitive performance and autonomic function following the ingestion of either, a vitamin complex with guarana, a caffeine dose alone at the same concentration than in guarana, and placebo. The main findings of the study were that (1) decisional cognitive performance was significantly improved from 30 min to 90 min after the ingestion of the complex with guarana and this change differed from that observed after a single dose of caffeine; (2) after ingestion of the complex with guarana, stability in parasympathetic modulation was observed during the first hour of the experiment, whereas a decrease was observed for placebo and caffeine conditions; (3) In contrast with previous studies examining the effect of caffeine ingestion on cognition, no effect was observed on SRT.

The improvement in decisional cognitive performance observed during the go/no-go task following guarana ingestion is in agreement with previous results reported in studies comparing guarana and placebo ingestion [3,4,5,6,7]. Indeed, Kennedy *et al.* [5] have observed that the ingestion of 75 mg of guarana in healthy subjects leads to an improvement in memory performance. Thereafter, Haskell *et al.* [6] compared four different doses (37.5 mg, 75 mg, 150 mg and 300 mg) of a standardized guarana extract. Results indicated that the two lower doses produced greater beneficial effects on memory performance than the higher doses. One interesting result of this study is that 75 mg of guarana contains a level of caffeine (9 mg) that is generally considered too low to affect cognitive performance. The authors proposed that the positive effect of guarana is not only related to caffeine. Furthermore, Campos *et al.* [27] have compared guarana (25 and 50 mg/kg) and caffeine (10 and 20 mg/kg) effects on mouse behavior. These authors reported that both guarana and caffeine have a significant effect on behavior, but suggested that the mechanisms underlying antidepressant-like activity are different. The fact that our results indicate a differentiated effect between guarana and caffeine ingestion supports this hypothesis. In our study the improvement in cognitive performance during the go/no-go task is only observed following guarana ingestion. Guarana contains 4% to 8% caffeine, as well as other alkaloids, such as theophylline (500–750 ppm) and theobromine (300–500 ppm), tannins or saponins. We assume that the difference between the complex ingestion and caffeine could be related to the presence of other psychoactive substances including tannins and saponins that are known to influence cognitive performance [13]. Vitamins present in the complex could also have a positive effect on cognitive performance. In elderly people with neurological diseases several minerals and vitamins have been classically described to positively affect cognitive performance. For example, a significant effect has been observed in elderly people of vitamin B, e.g., [28], or vitamins C and E on cognition, e.g. [29]. In a recent review, Huskisson *et al.* [30] indicated that vitamins B and C also present in the complex used in our study seem most effective, however, these authors also suggested that the results in this area need to be validated in healthy subjects.

In our study no effect of the ingestion of a complex with guarana or caffeine alone has been observed on SRT. This result is different from those reported in previous works showing a significant improvement in TRS after ingestion of caffeine without increasing the number of anticipated responses [31,32]. However, in all studies doses of caffeine were greater than those used in this study and positive effects were observed for a dose of caffeine of 200 or 250 mg, respectively, in studies conducted by Lieberman, *et al.* [32] and Attwood *et al.* [31]. Therefore, the dose of caffeine in the complex with guarana seems too low to induce an improvement in performance during perceptual-motor tasks involving low uncertainty, such as SRT.

In this study after either complex with guarana, stability in parasympathetic modulation (RMSSD or HF) was observed during the first hour after ingestion, whereas a decrease was observed for placebo and caffeine alone. Furthermore these effects are concomitant with changes in decision-making performance.

Heart rate variability is a noninvasive measure of autonomic contributions to cardiac functioning. The time domain (RMSSD) and the frequency domain (HF) reflect the parasympathetic modulation of the autonomic system even if they are not always strongly correlated [18]. Therefore a decrease in RMSSD and/or HF indicates a more sympathetic-and less parasympathetic-related modulation. Within this framework previous works have drawn links between HRV decrease and anxiety or worry [22,33]. Therefore the stability in parasympathetic modulation observed in our study indirectly suggests that ingestion of guarana complex does not lead to an increase in anxiety in our healthy subjects immediately after the ingestion.

Many investigations from cognitive psycho-physiologists have previously studied the relationship between HRV and cognitive performance, but results remain contradictory according to the nature of the cognitive task and there are still many questions regarding the relationship between acute changes in HRV and cognitive performance, e.g., [18]. On the one hand, results recorded during task engagement suggest that a pattern of cardiovascular adjustments, including enhanced sympathetic and reduced vagal cardiovascular influences, may induce an adaptive state associated with improved cognitive functioning and faster RT [19,34]. On the other hand, when HRV is assessed independent of task engagement, like in our study, a decrease in HRV is associated with worse cognitive performance [35]. Recent models from the literature suggest a link between HRV and prefrontal cortical activity involved in executive functions, such as decision-making. For example, the Neurovisceral Integration Model proposes that all these processes of cognitive, affective and physiological regulation may be related for goal-directed behavior [36]. Within this framework, low HRV values or a decrease in HRV could be viewed as reflecting anxiety and worry, but it could also reflect mental load [37,38] and inefficient or ineffective cognitive function [21,37]. The existing literature tends to yield supportive evidence, indicating that HRV is positively associated with performance during response inhibition and working memory tasks [20,35,39,40]. Thus, in our study the decrease in HRV in Pl and C conditions during the first hour of the experiment could be related to an impairment in cognitive performance and an increase in mental load [36], whereas the stability in HRV in Ac during more than one hour seems to indicate the efficacy of such ingestion to maintain parasympathetic modulation and cognitive performance during cognitive tasks requiring executive processes.

## 5. Conclusions

This research supports previous findings concerning the psychoactive properties of guarana and provides evidence that a multi-vitamin–mineral ingestion with added guarana improves decision-making performance without any additional impairment of the autonomic nervous systems’ regulation or side effects on anxiety.
